# Host Determinants of Reinfection with Schistosomes in Humans: A Systematic Review and Meta-analysis

**DOI:** 10.1371/journal.pntd.0003164

**Published:** 2014-09-11

**Authors:** Evaristus Chibunna Mbanefo, Nguyen Tien Huy, Anita Akpeedje Wadagni, Christine Ifeoma Eneanya, Obioma Nwaorgu, Kenji Hirayama

**Affiliations:** 1 Department of Parasitology and Entomology, Faculty of Bioscience, Nnamdi Azikiwe University, Awka, Nigeria; 2 Department of Immunogenetics, Institute of Tropical Medicine (NEKKEN), Nagasaki University, Sakamoto, Nagasaki, Japan; University of Queensland, Australia

## Abstract

**Background:**

Schistosomiasis is still a major public health burden in the tropics and subtropics. Although there is an effective chemotherapy (Praziquantel) for this disease, reinfection occurs rapidly after mass drug administration (MDA). Because the entire population do not get reinfected at the same rate, it is possible that host factors may play a dominant role in determining resistance or susceptibility to reinfection with schistosomes. Here, we systematically reviewed and meta-analyzed studies that reported associations between reinfection with the principal human-infecting species (*S. mansoni, S. japonicum* and *S. haematobium*) and host socio-demographic, epidemiological, immunological and genetic factors.

**Methodology/Principal Findings:**

PubMed, Scopus, Google Scholar, Cochrane Review Library and African Journals Online public databases were searched in October 2013 to retrieve studies assessing association of host factors with reinfection with schistosomes. Meta-analysis was performed to generate pooled odds ratios and standardized mean differences as overall effect estimates for dichotomous and continuous variables, respectively. Quality assessment of included studies, heterogeneity between studies and publication bias were also assessed. Out of the initial 2739 records, 109 studies were included in the analyses, of which only 32 studies with 37 data sets were eligible for quantitative data synthesis. Among several host factors identified, strong positive association was found with age and pre-treatment intensity, and only slightly for gender. These factors are major determinants of exposure and disease transmission. Significant positive association was found with anti-SWA IgG4 level, and a negative overall effect for association with IgE levels. This reconfirmed the concept that IgE/IgG4 balance is a major determinant of protective immunity against schistosomiasis. Other identified determinants were reported by a small number of studies to enable interpretation.

**Conclusions:**

Our data contribute to the understanding of host-parasite interaction as it affects reinfection, and is a potential tool to guide planning and tailoring of community interventions to target high-risk groups.

## Author Summary

One of the major challenges of schistosomiasis control is that disease prevalence reverts to baseline levels after mass drug administration due to high rate of reinfection. Host factors play a major role in determining resistance or susceptibility to reinfection with schistosomiasis and other diseases. We systematically searched and analyzed studies that identified potential host determinants of reinfection with schistosomes. Among demographic variables, age but not gender was strongly associated with reinfection with schistosomes. Pretreatment infection intensity was also identified as a major determinant of reinfection. Positive association with IgG4 levels and negative association with IgE levels reconfirmed the notion that IgE/IgG4 balance is the major factor controlling protective immunity against schistosomiasis. Other factors were reported by few studies to allow correct inferences. These results contribute to our understanding of host-parasite relationship as it affects reinfection, and will be useful for planning and targeting the limited resources for intervention on high-risk groups.

## Introduction

Schistosomiasis is still an important helminthic infection in terms of severe morbidity that can result as a consequence of infection. Over 200 million people are infected and more than 700 million people are still at risk of getting infected with schistosomiasis [1]. Although the disease can be effectively treated with Praziquantel, reinfection occurs rapidly after mass drug administration (MDA). An effective vaccine used singly or in combination with chemotherapy is the optimum approach [2]. However, such a vaccine is presently not available, although some candidates are still in the development pipeline. Since chemotherapy by MDA is presently the only available intervention in endemic areas, there is need to identify the host factors that increase susceptibility to reinfection with schistosomes for targeted intervention in high-risk groups.

In addition to the demographic, socioeconomic and epidemiological variables that may predispose certain subset of the population to reinfection, several human studies in endemic areas have provided insight into the potential resistance inducing immune response phenotypes [2]–[8]. Many of these studies have found associations between reinfection with schistosomes and IgE/IgG4 balance. Schistosomiasis has previously been shown to be under the control of the cytokine genes cluster on chromosome 5q31-q33 region, called SM1 [7], [9], [10]. It is also possible that several other immunogenetic factors in addition to this cytokine genes cluster, including the genes controlling IgE levels, may be associated with reinfection with schistosomiasis [11]–[15]. However, it remains to be determined whether variations in these genes or which of the variations in these genes are potential determinants of reinfection.

We undertook this meta-analysis to identify and describe studies that had identified host determinants, including socio-demographic, epidemiological and immunogenetic factors that are associated with reinfection with schistosomes.

## Methods

### Protocol registration

This study was performed in accordance with the recommendations of the PRISMA statement [16], [17]. This statement summarized in the PRISMA 2009 checklist is supplied as supplementary information (Figure S1). The protocol for this study was determined prior to commencement of the study, and was registered in PROSPERO-International prospective register of systematic reviews with identification number CRD42013006582 available from http://www.crd.york.ac.uk/PROSPERO/display_record.asp?ID=CRD42013006582.

### Eligibility criteria

Studies that assessed host factors of reinfection with schistosomes were included in this review. While all eligible studies on all identified host determinants were included in the qualitative systematic review, only factors reported by more than one study and whose data can be reliably extracted were included in the quantitative meta-analysis. All relevant studies were included irrespective of study type, study design, language and date. We limited included studies to studies performed on human subjects. Reports were excluded if the reported information was on a study performed on animals, if they were case studies, correspondence or reviews, and if the data could not be reliably retrieved. Decisions on eligibility were made by two independent reviewers and all discrepancies and disagreements as regards study and report eligibility were resolved by discussion or consensus with a third reviewer, when necessary.

### Information source

Studies analyzed in this review were identified by searching electronic public databases including: PubMed (http://www.ncbi.nlm.nih.gov/pubmed), Scopus (http://www.scopus.com/), Google Scholar (http://scholar.google.com/), Cochrane Review Library (http://www.cochrane.org/cochrane-reviews) and African Journals Online (AJOL) (http://www.ajol.info/index.php/index/search). The searches were performed in October 2013 with no limit set for the dates of publications. Reference lists of eligible articles were also checked to obtain supplementary information and records of potentially relevant studies and reports. Efforts were made to contact authors for full texts, clarification on data and for supplementary data, when necessary. When responses with the necessary details were not received from authors during the duration of the study and after two reminders, such studies were excluded and classified as “full text not available”.

### Search strategy

Initial searches were performed on PubMed and Scopus databases using the broad search term: “((reinfection OR re-infection OR resistance OR resistant OR susceptibility OR susceptible OR haplotype OR allele OR SNP OR “single nucleotide polymorphism” OR variant OR polymorphism OR “genetic factors” OR HLA OR “human leucocyte antigen”) AND (schistosom* OR bilharzi*))” to retrieve socio-demographic, epidemiological and immunogenetic factors. For Advanced Google Scholar, we filled in the term “schistosoma OR schistosomiasis OR schistosome OR bilharzia OR bilharziasis” in the field “with all of the word”, and the words “reinfection OR re-infection OR resistance OR resistant OR susceptibility OR susceptible OR “host factors” OR “genetic factors” OR haplotype OR allele OR SNP OR “single nucleotide polymorphism” OR variant OR polymorphism, in the field “with at least one of the words” to search the titles of articles in Google scholar database. The Cochrane Library and African Journals Online databases were searched with the broad term “schistosoma OR schistosomiasis OR bilharzia OR bilharziasis”.

### Study selection

Two independent reviewers performed initial eligibility assessments on the retrieved titles and abstracts, for inclusion in the systematic review. Full texts of eligible articles were then retrieved and reviewed for inclusion in the systematic review, and further screened for inclusion in the meta-analysis using the inclusion criteria. In both steps of the screening, inclusion or exclusion of a study by both reviewers was considered conclusive, while inclusion or otherwise of studies judged eligible, controversial or ambiguous by either of the reviewers was resolved by discussion and consensus between the two reviewers. When necessary, disagreements and discrepancies were resolved by consensus with a third reviewer. Care was taken to identify more than one report describing a single study. When such was encountered, the overlap was identified and resolved, with contacts made to the authors when necessary. The study selection procedure was summarized in a systematic review flow chart.

### Data collection process and data items

We adopted the methodology and data extraction template of The Review Manager (RevMan v5.2) from The Nordic Cochrane Centre, Cochrane Collaboration, 2012 for data extraction, in addition to other relevant data items as determined by the reviewers. As much as possible, the following pieces of information were obtained from eligible study reports by two independent reviewers, in a non-blinded manner: study ID (lead author name and year), study type, study period, study location, problem addressed (reinfection with schistosomes), species studied, host factors assessed, study aim, recruitment, inclusion, exclusion, informed consent, ethical approval, number of participants, study completion rate, statistical methods and funding. Given that the host factors we identified and reviewed were not set *a priori*, the factors were included on first observation. Thus, a study assessing several host factors was included respectively in the meta-analysis for each of the factors; while overlap from multiple reports referring to a single study was resolved to avoid duplication. When studies in different locations were separately reported in a single article, the study was included twice for each of the areas differentiated using footnotes.

### Quality assessment of included studies

To assess risk of bias within selected studies, we adopted the quality assessment tool in the Cochrane RevMan v5.2 program. Briefly, this method takes into account four factors: selection bias by evaluating the sampling and randomization procedure, performance bias by assessing the level of blinding of personnel, detection bias by evaluating the level of blinding of outcome assessment, and reporting bias by assessing the presence of selective reporting in data presentation. We created a quality scoring system based on these RevMan v5.2 quality assessment items, with levels of risk of bias scored as “−1” for high risk, “0” for unclear risk and “1” for low risk of bias. These parameters (n = 4), in addition to availability of descriptions of (n = 8): host determinants, outcomes definitions, inclusion criteria, exclusion criteria, method of diagnosis, mass chemotherapy, confirmation of cure prior to inclusion and follow-up period, were used to create a quality score based on 12 items on a scale of 100% for each included study (Table S1).

### Assessment of risk of bias across studies

To assess the risk of bias across studies, Begg's funnel plots were generated to assess publication bias across the reviewed studies [18], [19]. Funnel plots were created for each factor by plotting the effects measure (odds ratio) against the standard error of its logarithm. The symmetries of the funnel plots were first assessed visually. When potential publication bias was identified, the trim and fill method proposed by Duvall and Tweedie [20] was applied. No further test of bias or symmetry of the funnel was performed since no publication bias was apparent.

### Definition of outcomes and risk factors

The outcomes definitions were pre-determined by the reviewers, and all included studies sufficiently satisfy these criteria. Briefly, “resistance to reinfection” was defined as absence of parasite egg in multiple parasitological examinations after treatment (and confirmation of cure), followed by a follow-up period (for uniformity, data on ∼12 months follow-up were pooled) despite exposure to the parasite. Conversely, “susceptibility to reinfection” was defined as positive parasitological examination within 6 to 12 months after chemotherapy and cure. Among the risk factors identified, we adopted <10 years old as the definition for younger children. This was because all the included studies defined younger children as either <9 or <10 years old, apart from three studies that defined younger children as <13, <14 and <15 years old, respectively (Table S1). High (moderate to high) pretreatment infection intensities was defined as >50 eggs/10 ml of urine for *S. haematobium*, and >100 eggs/g of feces for *S. mansoni* and *S. japonicum*. For antibody levels, only data from studies utilizing the predetermined established method (ELISA) were pooled. While slight variations exist in the methods adopted in each study for estimation of antibody levels, these were ignored since similar conditions apply for the comparator in each study. Only data from studies satisfying these definitions were pooled in data synthesis.

### Quantitative data synthesis (meta-analyses)

Data from eligible studies were combined using meta-analysis performed on The Review Manager (RevMan v5.2) from The Nordic Cochrane Centre, Cochrane Collaboration, 2012 [21]. We meta-analyzed and interpreted all host factors reported in more than one studies without setting any cut off for the minimum number of studies required for valid interpretation. For each identified host factor reported as dichotomous outcome, 2×2 contingency tables were generated and the odds ratio (OR) with the corresponding 95% confidence intervals (95% CI) were calculated. For studies reporting continuous outcomes (such as antibody levels), the input data was mean and standard deviation (SD) with the standardized mean difference (SMD) as the effect measure. When the standard deviation was not reported, it was computed with the calculator function in RevMan v5.2, using other supplied data (e.g. mean, SEM, *p*-value etc.). For each factor analyzed, a forest plot showing the respective odds ratios or standardized mean differences with their corresponding 95% confidence interval for each study and for the pooled data were generated. The test of overall effect was assessed using the Z-statistics on RevMan v5.2 with statistical significance set at *p*<0.05. Subgroup analysis based on species studied was performed when necessary, especially for host factors with sufficient number of included studies.

### Test of heterogeneity between studies

Heterogeneity (inconsistency) between studies was evaluated using the Cochrane Q (*Chi^2^* test) and *I^2^* statistics in RevMan v5.2 [22]. The statistical significance for heterogeneity using the *Chi^2^* test was set as *p*<0.10. Estimates of degree of heterogeneity using *I^2^* were made by setting 25%, 50%, or 75% as limits for low, moderate or high heterogeneity, respectively [22]. The fixed-effects model with weighting of the studies was used when there was a lack of significant heterogeneity (*p*>0.10), while the random-effects model with weighting of the studies was used when there was heterogeneity between studies (*p*<0.10) and *I^2^* values of over 50%. A major drawback of the random-effects model is that it assigns relatively equal weight to studies. Therefore, fixed-effects model was preferred over random-effects, although random-effects model was still applied when significant heterogeneity was recorded between studies.

### Sensitivity analysis

For sensitivity analysis, we adopted the methods recommended for Cochrane systematic reviews. Each meta-analysis of the association of reinfection with a host factor was reanalyzed with the exclusion of each individual study to examine the effect of a single study on the outcome of meta-analysis. In addition, to examine the effect of the largest and smaller studies on the outcome of the meta-analysis, cumulative meta-analysis was performed with studies ordered according to the sample size. Also, sensitivity testing to identify the effect of subgroups was performed by subgroup analysis. This was achieved by comparing the results of the meta-analysis after exclusion of each subgroup.

## Results

### Study selection

**Figure 1:**
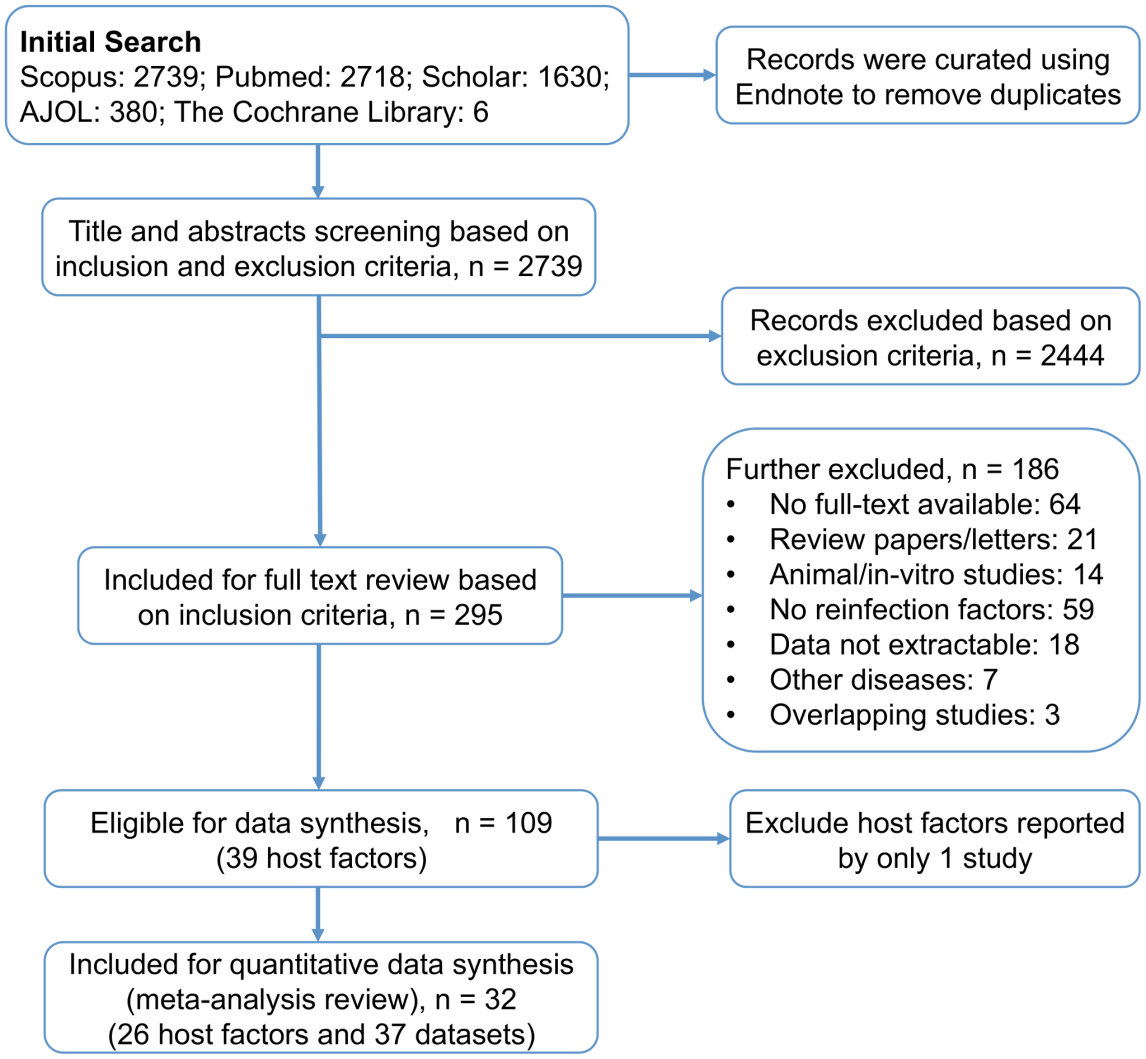
Flow diagram for the search and systematic review process.

Using the broad search terms, initial screening of public databases yielded 2739 study reports. Out of these studies, 295 were included for full text reading based on initial title and abstract screening using the inclusion criteria. The two reviewers agreed with 284 decisions and 11 discrepancies were resolved by discussion and consensus. For some reasons that are outlined in Figure 1, further 186 study reports were excluded and a total of 109 studies identifying 39 host factors were included for the data synthesis. However, some of the identified host determinants were reported by only 1 study and were further excluded in the final meta-analysis. Finally, 32 study reports on 26 host determinants of reinfection were included in the final quantitative data synthesis (meta-analysis). Five of these study reports were on two independent data sets [23]–[27], thus, a total of 37 datasets were included in the meta-analysis (Figure 1).

### Characteristics of included studies

The characteristics of the 32 studies included in the meta-analysis were fully described in Table S1. This table outlined the study ID, study location, study period, sample size, gender ratio, age range and species studied. Out of the 32 included studies; 13 were on reinfection with *Schistosoma mansoni*, 7 were on *S. japonicum*, 10 studies assessed reinfection with *S. haematobium*, and 2 studies were on both *S. mansoni* and *S. haematobium*. Based on the population of subjects studied; 20 studies were on both children and adults, 11 studies were on children alone, while only 1 study was on adult subjects alone. All included studies were cohort studies (n = 32), with prospective data collection method (Table S1).

### Quality assessment of included studies

Assessment of risk of bias within selected studies was performed using the quality assessment tool in Cochrane RevMan v5.2 program, modified as detailed in the Method section. The result of the quality assessment based on the 12 items on a scale of 100% showed that only 2 studies scored the maximum points (100%). The other included studies scored over 75% points in the study quality assessment, indicating their suitability for inclusion in the meta-analysis (Table S1).

### Synthesis of results and meta-analysis

A total of 39 host factors comprising socio-demographic, epidemiological, immunological, genetic variants and other variables were identified from the included studies. However, 13 of these host determinants were reported in only one study and were subsequently excluded in the meta-analysis. The full list of 39 identified host factors with their corresponding statistics and effect estimates were included as supplementary information (Table S2). The summary of the meta-analyses on 26 host factors, including: the number of pooled studies, data analyses model adopted, the tests of heterogeneity, association analyses and the study references were presented in Table 1. Several demographic, epidemiological and immunological variables were identified, with some of the variables showing strong association with reinfection with schistosomes. These are further described in subsequent sections.

**Table 1:** Host determinants of reinfection with schistosomes identified in this study.

Host Factors	No Reps	Model	Heterogeneity		Association		Study References
			*X^2^* (*p*-val.)	*I^2^*	OR/SMD (95% CI)	Z (*p*-val.)	
**1. Demographic factors**							
Age (<10)	19	Rand.	*p*<0.0001	74	1.91 [1.41, 2.60]	*p*<0.0001	[24], [26], [28]–[42]
Gender (M)	20	Rand.	*p*<0.0001	88	1.45 [1.02, 2.05]	*p* = 0.04	[24]–[26], [28]–[32], [34]–[36], [39], [40], [42]–[46]
**2. Epidemiological factors**							
PTI	7	Rand.	*p* = 0.006	67	2.85 [1.97, 4.12]	*p*<0.0001	[26], [27], [30], [36], [40]
Exposure	4	Rand.	*p*<0.0001	91	2.34 [0.93, 5.85]	*p* = 0.07	[36], [40], [42], [44]
HTA	5	Rand.	*p*<0.0001	91	2.24 [0.63, 7.91]	*p* = 0.21	[37], [42], [44], [47]
**3. Antibodies**							
· **IgE**							
SWA	7	Rand.	*p*<0.0001	88	−0.06 [−0.59, 0.46]	*p* = 0.82	[23], [31], [39], [44], [49], [53]
SEA	8	Rand.	*p* = 0.001	71	−0.03 [−0.38, 0.32]	*p* = 0.88	[23], [31], [39], [44], [48]–[51]
· **IgG4**							
SWA	5	Fixed	*p* = 0.38	5	0.47 [0.26, 0.68]	*p*<0.0001	[23], [31], [39], [44], [49]
SEA	9	Rand.	*p*<0.0001	89	0.41 [−0.13, 0.95]	*p* = 0.14	[23], [31], [39], [44], [48]–[51]
· **IgG1**							
SWA	4	Rand.	*p*<0.0001	89	0.71 [0.06, 1.37]	*p* = 0.03	[23], [31], [45], [49]
SEA	5	Rand.	*p* = 0.005	73	0.56 [0.10, 1.03]	*p* = 0.02	[23], [31], [48], [49], [51]
· **IgG2**							
SWA	3	Rand.	*p*<0.0001	90	0.67 [−0.15, 1.49]	*p* = 0.11	[23], [31], [49]
SEA	4	Rand.	*p*<0.0001	92	0.87 [0.02, 1.71]	*p* = 0.04	[23], [31], [49], [51]
· **IgG3**							
SWA	2	Rand.	*p* = 0.04	77	−0.22 [−0.85, 0.42]	*p* = 0.51	[31], [49]
SEA	3	Fixed	*p* = 0.70	0	0.04 [−0.21, 0.29]	*p* = 0.77	[31], [49], [51]
· **IgA**							
SWA	3	Rand.	*p*<0.0001	95	0.50 [−0.67, 1.67]	*p* = 0.40	[23], [31], [49]
SEA	5	Rand.	*p*<0.0001	93	0.54 [−0.42, 1.50]	*p* = 0.27	[23], [31], [48], [49], [51]
· **IgM**							
SWA	3	Rand.	*p*<0.0001	98	1.84 [−1.11, 4.79]	*p* = 0.22	[23], [31], [49]
SEA	3	Rand.	*p*<0.0001	96	1.19 [−0.50, 2.89]	*p* = 0.17	[23], [31], [51]
**4. Cytokines**							
IFN-g	4	Fixed	*p* = 0.36	1	−0.22 [−0.52, 0.08]	*p* = 0.15	[6], [44], [45], [48]
IL-10	4	Fixed	*p* = 0.08	56	−0.15 [−0.44, 0.13]	*p* = 0.29	[6], [44], [45], [48]
TNF-a	2	Rand.	*p* = 0.03	79	−0.27 [−0.77, 0.22]	*p* = 0.28	[6], [51]
IL-5	3	Rand.	*p* = 0.004	82	−0.17 [−1.38, 1.04]	*p* = 0.78	[6], [44], [48]
**5. Immune cell surface marker**							
CD4	2	Fixed	*p* = 0.25	23	−0.62 [−1.05, −0.18]	*p* = 0.005	[6], [81]
CD8	2	Rand.	*p* = 0.003	89	0.08 [−1.71, 1.86]	*p* = 0.93	[6], [81]
CD19	2	Fixed	*p* = 0.40	0	0.38 [−0.04, 0.81]	*p* = 0.08	[6], [81]

NB: No = Number of included studies; OR = odds ratio; SMD = standardized mean difference; *p*-val. = *p*-value; PTI = Pre-treatment intensity of infection; Exposure = Exposure rate; HTA = High transmission area; SWA = Schistosoma adult worm antigen; SEA = Schistosoma egg antigen.

### Association of demographic factors with reinfection with schistosomes

#### 1. Age (<10 years old)

**Figure 2:**
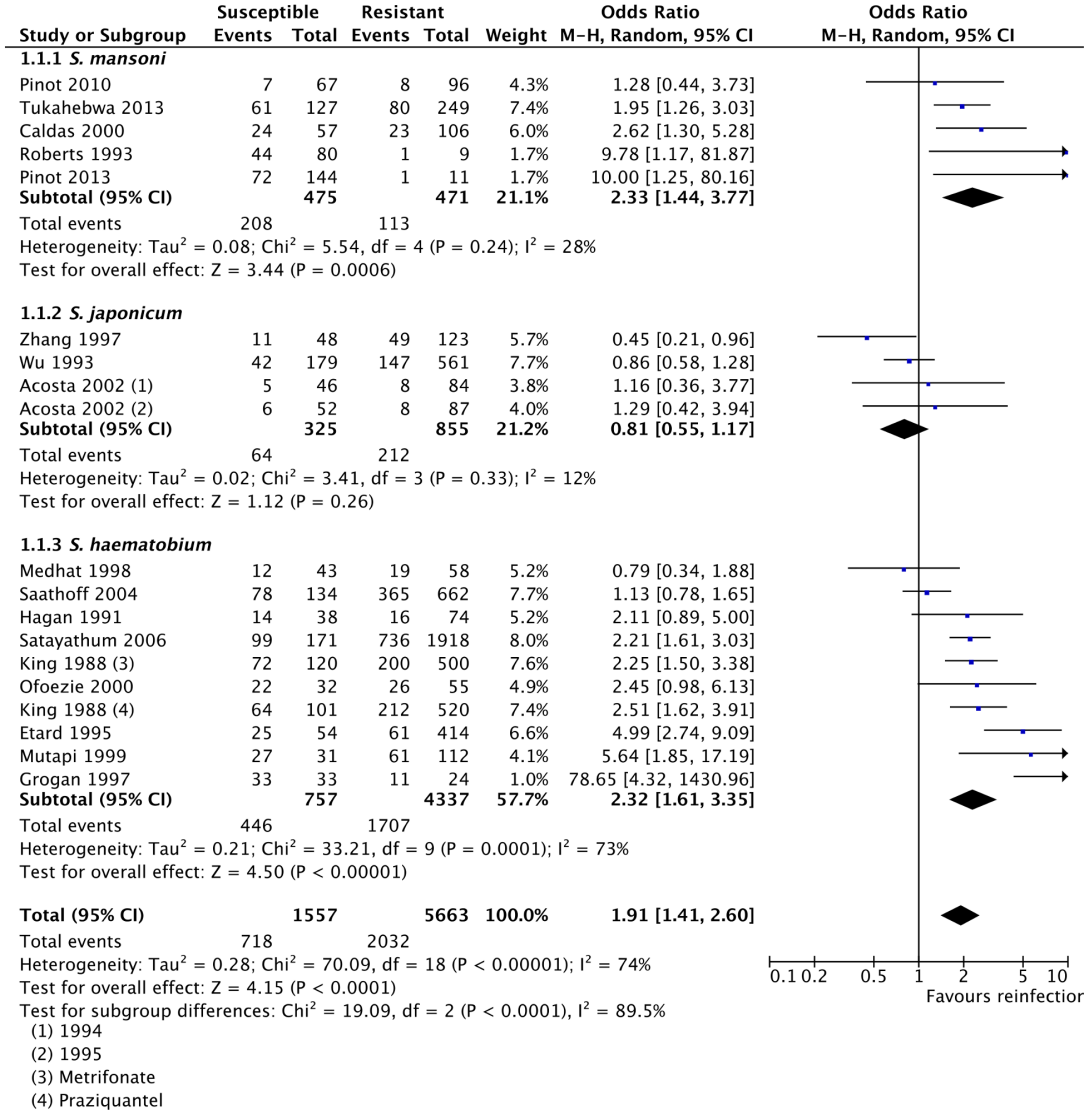
Association of younger age (<10 years old) with reinfection with schistosomes. Presented here is the meta-analysis forest plot showing the pooled odds ratio and the corresponding 95% CI, subgroup analysis by species, and assessment of heterogeneity among studies. There was a strong statistically significant positive association between younger age (<10 years old) and reinfection with schistosomes.

Age was identified as a major factor that may predispose certain subsets of the population to reinfection with schistosomes. In this review, we assessed the odds of reinfection among children less than 10 years old as compared to the rest of the population (Figure 2). Significant heterogeneity was observed among the included studies (*p*<0.0001, *I^2^* = 74%), therefore, random effects model was applied for the meta-analysis. This heterogeneity was probably due to the variability of the studied populations. While some of the studies were on school-aged children (5–18 years range), others included the whole population (Table S1). Pooled odds ratio showed that younger age (<10 years old) was positively associated with reinfection with schistosomes (OR = 1.91, 95% CI = 1.41–2.60, Z = 4.15, *p*<0.0001) (Figure 2).

Sensitivity analysis by analysis of subgroups based on the species studied showed that while reinfection with *S. mansoni* [28]–[32] and *S. haematobium* [26], [33]–[40] showed strong association with age, such association was not observed when only studies on *S. japonicum* [24], [41], [42] were considered (Figure 2). The positive association between reinfection and age (<10 years old) lost its statistical significance (*p* = 0.13) when all the studies on *S. haematobium* subgroup were excluded from the meta-analysis (Table S3). Also, sensitivity analysis by exclusion of a single study from the analysis (Table S3) or cumulative meta-analysis (Table S4) showed that the result of the meta-analysis was robust as the inclusion or exclusion of any single study did not affect the outcome of the odds ratio, Z-score and *p*-value.

#### 2. Gender (male)

**Figure 3:**
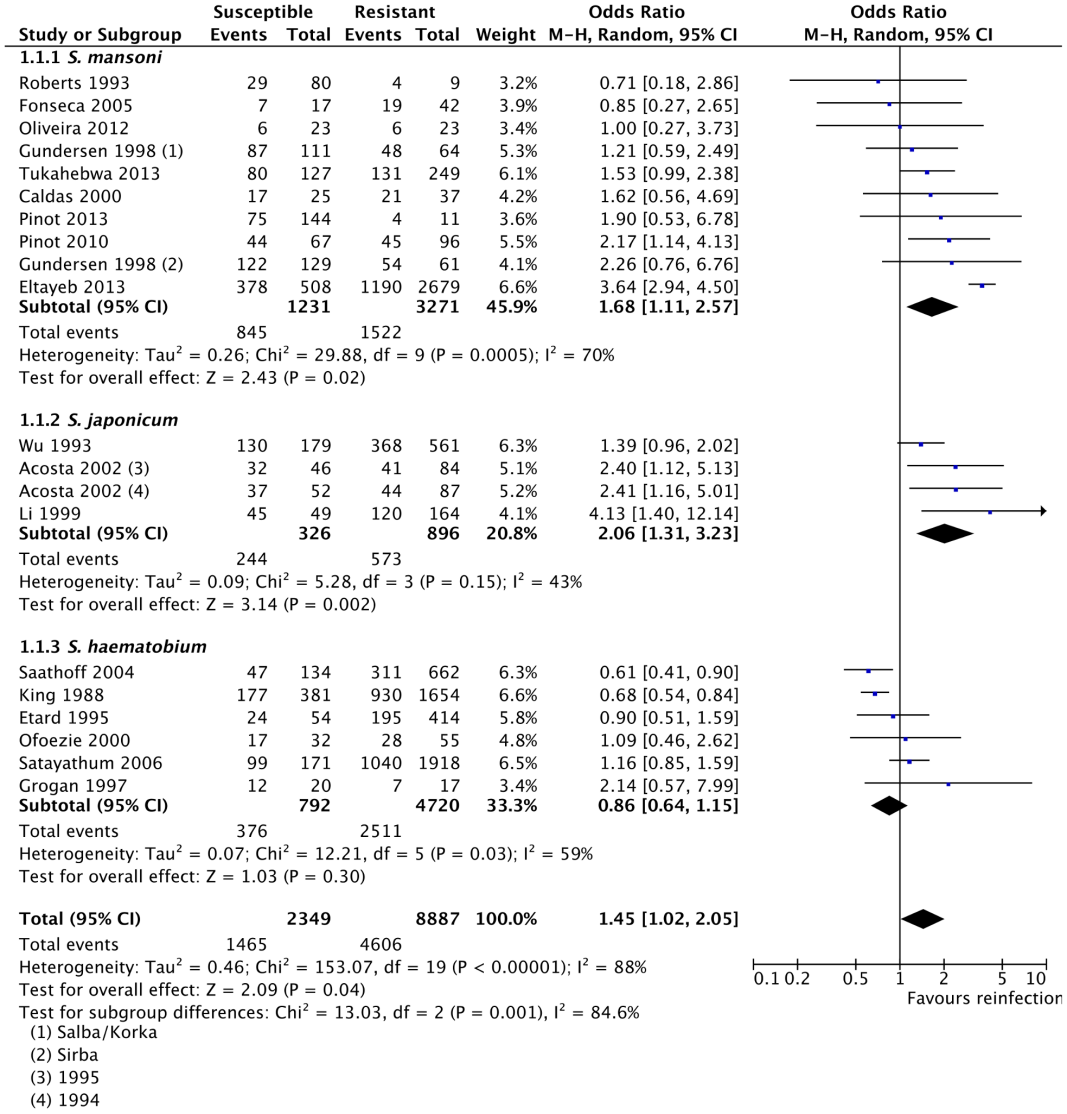
Association of gender (male) with reinfection with schistosomes. Presented here is the meta-analysis forest plot showing the pooled odds ratio and the corresponding 95% CI, subgroup analysis by species, and assessment of heterogeneity among studies. The observed positive association between reinfection and gender was only slightly significant.

We also assessed whether gender (being male) was a predisposing factor of reinfection with schistosomes. Because there was significant heterogeneity among the studies (*p*<0.0001, *I^2^* = 88%), random effects model was applied. Pooled odds ratio showed that although there was a positive association of male gender and reinfection with schistosomes, the association was only slightly statistically significant (OR = 1.45, 95% CI = 1.02–2.05, Z = 2.09, *p* = 0.04). Sensitivity analysis by subgroup analysis based on the species studied showed that statistically significant positive association was observed for association of male gender with reinfection with *S. mansoni* (*p* = 0.02) [25], [28]–[32], [43]–[45] and *S. japonicum* (*p* = 0.002) [24], [42], [46], while the association for reinfection with *S. haematobium* [26], [34]–[36], [39], [40] was not statistically significant (*p* = 0.30) (Figure 3). The exclusion of *S. mansoni* or *S. japonicum* subgroups in the meta-analysis yielded effect measures which were not statistically significant (*p* = 0.22 and *p* = 0.23, respectively). Conversely, the exclusion of data from *S. haematobium* subgroup significantly affected the result of the meta-analysis (OR = 1.85, 95% CI = 1.34–2.55, Z = 3.76, *p* = 0.0002) (Table S3). Furthermore, sensitivity analyses by exclusion of single studies (Table S3) and cumulative meta-analysis (Table S4) showed that two large studies [26], [34] significantly affected the result of the pooled effect estimate (Z-score and *p*-value).

### Association of epidemiological factors with reinfection with schistosomes

**Figure 4:**
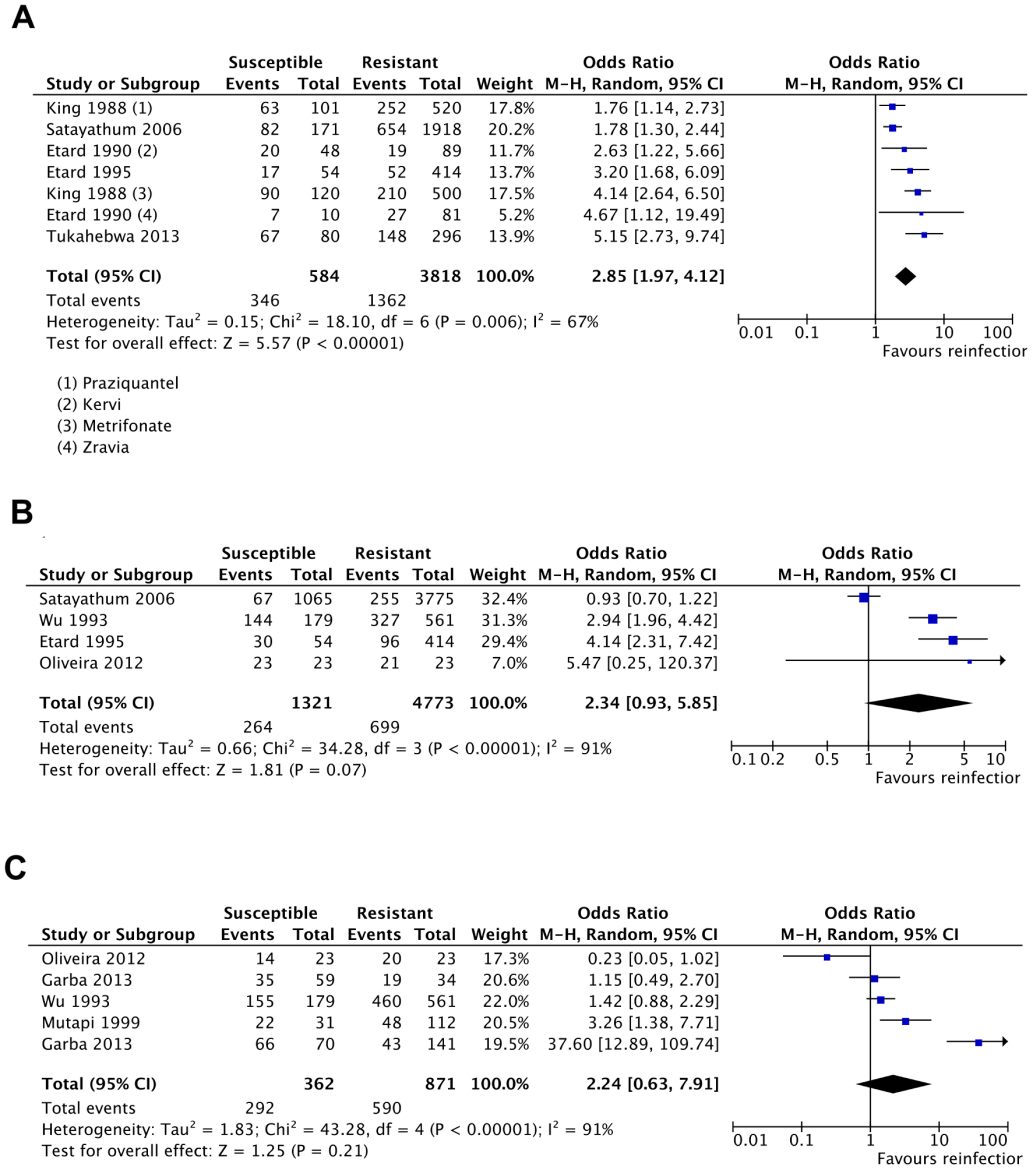
Association of epidemiological factors with reinfection with schistosomes. (**A**) Meta-analysis forest plot for the association of reinfection with high pretreatment intensity showing positive association with reinfection. (**B**) Forest plot for the association of reinfection with high rate of exposure showing association with reinfection without statistical significance. (**C**) Meta-analysis forest plot for the association of reinfection with residence in high transmission area did not show statistically significant association with reinfection.

Three major epidemiological factors of reinfection were identified, including: pre-treatment infection intensity [26], [27], [30], [36], [40], rate of exposure [36], [40], [42], [44] and levels of transmission in the studied area [37], [42], [44], [47]. There was strong positive association between high (moderate to high) pretreatment infection intensities (>50 eggs/10 ml of urine for *S. haematobium*, and >100 eggs/g of feces for *S. mansoni* and *S. japonicum*) and reinfection with schistosomes (OR = 2.85, 95% CI = 1.97–4.12, Z = 5.57, *p*<0.0001). (Figure 4A). The positive association between high rates of exposure (we defined high rate of exposure as “above average” exposure rate for a specific population as determined by authors) and reinfection with schistosomes was not statistically significant (OR = 2.34, 95% CI: 0.93–5.85, Z = 1.81, *p* = 0.07) (Figure 4B). Although there was also a positive correlation between residence in high transmission area and reinfection, the association was not statistically significant (OR = 2.24, 95% CI = 0.63–7.91, Z = 1.25, *p* = 0.21) (Figure 4C).

### Association of immunological factors with reinfection with schistosomes

**Figure 5:**
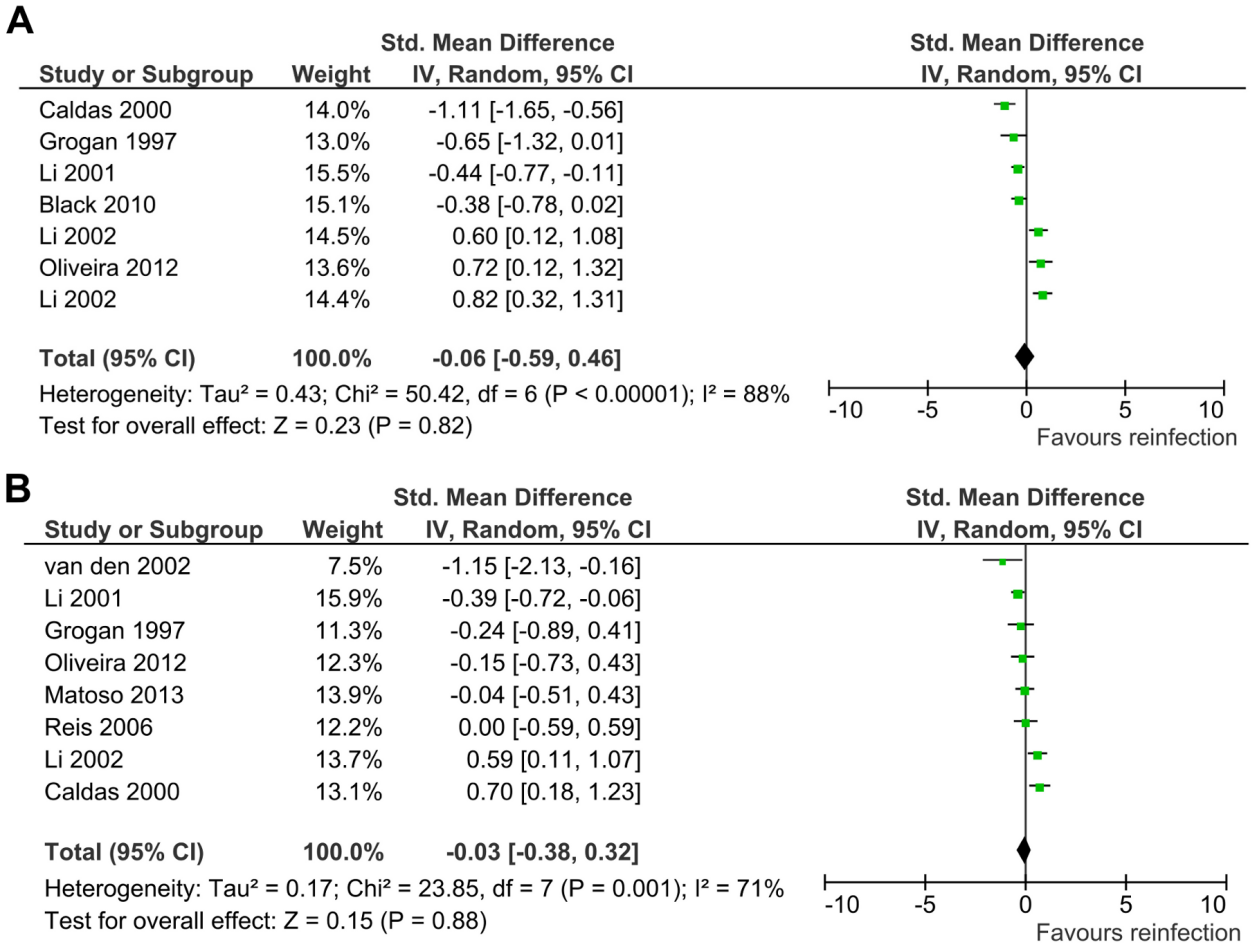
Association of IgE levels with reinfection with schistosomes. (**A**) Meta-analysis forest plot for the association of reinfection with anti-SWA IgE levels showing the pooled standardized mean difference and the corresponding 95% CI and assessment of heterogeneity among studies. The observed negative overall effect was not statistically significant. (**B**) Meta-analysis forest plot for the association of reinfection with anti-SEA IgE levels showing the pooled standardized mean difference and the corresponding 95% CI and assessment of heterogeneity among studies. The observed negative overall effect was not statistically significant.

Among the immunological factors identified in this review, most studies reported association between humoral responses and probability of reinfection with schistosomes. An interesting finding was a negative standardized mean difference observed in the association between IgE levels and reinfection with schistosomes (Figure 5 and Figure S2) inferred from meta-analysis on 8 studies [23], [31], [39], [44], [48]–[51]. However, subgroup analyses of these associations with IgE levels against adult worm antigen (SWA) (Figure 5A) and egg antigen (SEA) (Figure 5B) were not statistically significant (For anti-SWA IgE, SMD = −0.06, 95% CI = −0.59–0.46, Z = 0.23, *p* = 0.82; for anti-SEA IgE, SMD = −0.03, 95% CI = −0.38–0.32, Z = 0.15, *p* = 0.88). Sensitivity analysis by exclusion of individual studies showed that the exclusion of any of the included studies in this meta-analysis did not affect the pooled effect estimates (Table S3).

**Figure 6:**
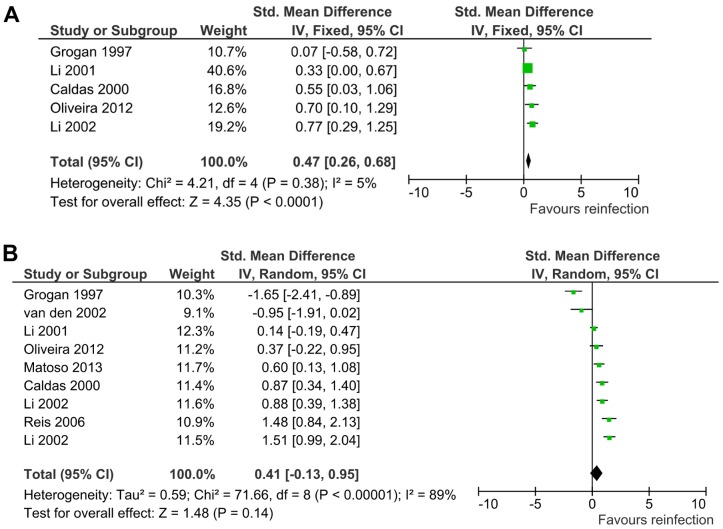
Association of IgG4 levels with reinfection with schistosomes. (**A**) Meta-analysis forest plot for the association of reinfection with anti-SWA IgG4 levels showing the pooled standardized mean difference and the corresponding 95% CI and assessment of heterogeneity among studies. IgG4 level was highly significantly associated with reinfection with schistosomes. (**B**) Meta-analysis forest plot for the association of reinfection with anti-SEA IgG4 levels showing the pooled standardized mean difference and the corresponding 95% CI and assessment of heterogeneity among studies. The observed positive overall effect was not statistically significant.

Conversely, strong positive association was observed between IgG4 levels and reinfection with schistosomes (Figure 6 and Figure S2). However, while the association between reinfection and anti-SWA IgG4 levels was statistically significant (SMD = 0.47, 95% CI = 0.26–0.68, Z = 4.35, *p*<0.0001) (Figure 6A); the association between reinfection and anti-SEA IgG4 levels was not statistically significant (SMD = 0.41, 95% CI = −0.13–0.95, Z = 1.48, *p* = 0.14) (Figure 6B). Sensitivity analysis showed that while the exclusion of any single study did not affect the result of the association between reinfection with schistosomes and anti-SWA IgG4 levels, the exclusion of one study [39] resulted in positive association with statistical significance for the association of anti-SEA IgG4 with reinfection with schistosomes (Table S3).

Equally, positive associations were observed for association of reinfection with schistosomes with levels of IgG1, IgG2, IgA and IgM against SWA (Figure S3A, B, D and E) and SEA (Figure S4A, B, D and E); while negative association was observed between reinfection and levels of IgG3 (Figure S3C and Figure S4C). However, there were limited number of studies reporting these factors and the associations were not statistically significant except for IgG1.

Some cellular immune response factors were also identified. However, there were consistently small number of studies reporting these factors, and the associations were not statistically significant (Table 1 and Table S2). We observed negative standardized mean differences from the associations of reinfection with levels of IFN-γ, IL-5, IL-10, IL-13, TNF-α and CD4^+^ T-helper cells; and positive effect estimates from the association of reinfection with proportions of CD8, CD19 and CD16 positive cells. Other factors including the HLA gene polymorphisms, levels of total protein, albumin, total cholesterol, low-density lipoproteins and very low-density lipoproteins were also identified. However, these factors were all reported by only one study (Table S2).

### Risk of bias across studies

**Figure 7:**
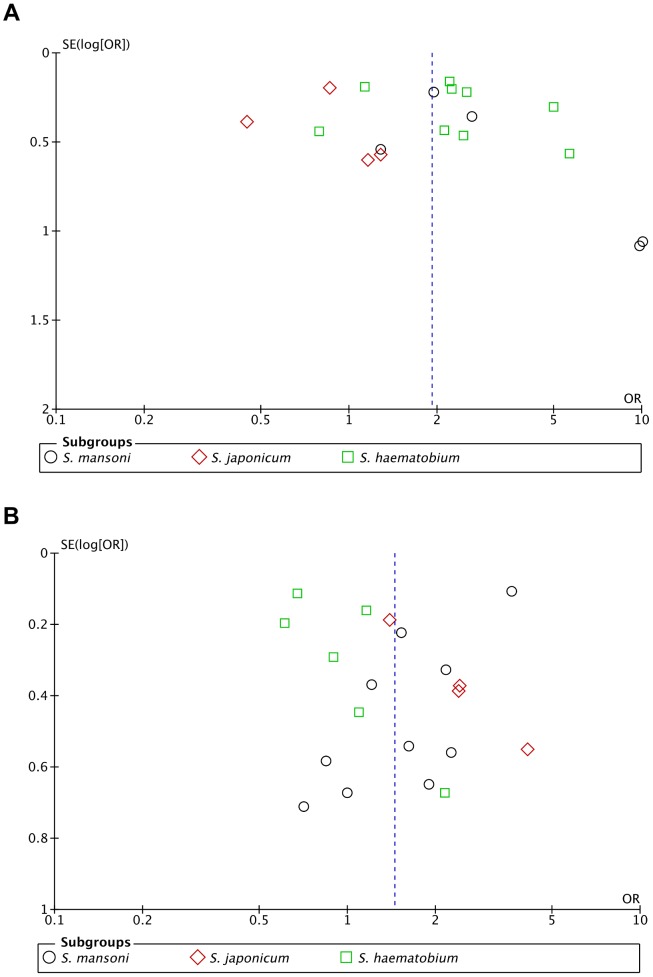
Funnel plots for assessment of publication bias. (**A**) Funnel plots for assessment of publication bias for studies assessing association of reinfection with age showing lack of significant publication bias in the included studies. (**B**) Funnel plots for assessment of publication bias for gender.

To assess outcome reporting bias and publication bias across studies, we generated funnel plots for two representative host factors with sufficient number of included studies (age and gender) by plotting the odds ratio (OR) on the x-axis, and the standard error of the log of odds ratio (SE(log[OR]) on the y-axis (Figure 7). The funnel plot showed the typical cone appearance with good symmetry, with the studies apparently distributed on either side of the pooled outcome effect estimate (Figure 7). Two outlier studies [29], [32] observed from the association of reinfection with age were of significantly small size (Figure 7A). To exclude potential small study effect, these studies were excluded and their exclusion did not affect the result of the pooled effect size. Equally, we also applied the trim and fill method proposed by Duvall and Tweedie [20] by adding studies equivalent to these outliers, which appear to be missing. Again, this did not affect the symmetry and the result of the pooled effect estimate. Also, subgroup analysis showed that all studies on *S. japonicum* were on the left side of the cone unlike the other species. However, exclusion of this subgroup (Table S3), or trim and fill method did not affect the results of the combined effect estimates. These indicate that there is minimal publication bias in these studies and no further test of bias was carried out.

## Discussion

Identification of host factors that predispose certain subsets of the population to reinfection with schistosomes, and indeed any other disease, is a major strategy that will guide planning and tailoring of community interventions to target high-risk groups. It is also important for targeting health education and limited resources for disease prevention. Our meta-analysis has identified some of the host determinants of reinfection with schistosomes. The outcomes showed strong positive association with age, and pretreatment intensity of infection, and only slight association with gender. Also, the IgE/IgG4 balance, which is well recognized as a major determinant of reinfection [2]–[5], was again reconfirmed by our meta-analysis. Our results showed that younger age and pretreatment intensity of infection, which are connected with behavioral differences in population subsets and disease transmission, play predominant role in determining the probability of reinfection with schistosomes. This is due to differences in rate of exposure, but not necessarily absence of protective immunity [52]. Conversely, the immunological parameters related with protective immunity, which may itself be associated with age and accumulated experience [40], [52]–[56]; also play major role in determining protection from infection but not exposure to the pathogen. It can therefore be inferred that exposure and age-related factors play a predominant role in disease transmission, while immunological factors control protective immunity against reinfection.

Younger age was positively associated with the rate of reinfection. Schistosomes are transmitted through skin penetration by the infective cercariae during water contact activities. Given that younger children are mainly involved in such high-risk water contact activities like domestic chores and recreation, this result is expected and is consistent with the results of other big intervention studies [26], [30], [34], [36]. However, species based subgroup analysis did not show positive association between age and reinfection with *S. japonicum*. It is not clear whether this is related to differences in the study cultural settings since unlike *S. haematobium* and *S. mansoni*; the distribution of *S. japonicum* is limited to South East Asia. However, limited number of studies assessed reinfection with *S. japonicum*, and unlike studies on the other species which sometimes involved only children, all studies on *S. japonicum* involved both adults and children; a major source of heterogeneity among the included studies. Also, *S. japonicum* has some peculiarities that may also contribute to the observed heterogeneity, including: its zoonotic nature, the generally much lower prevalence (especially in recent decades) and the fact that exposure is often mainly during occupational activities instead of domestic and recreational ones.

Our analyses showed only slight association between gender and reinfection with schistosomes. Although boys and girls may have major behavioral but not biological differences that can affect rate of reinfection [28], this distinction is very minimal among the younger age group. Even among the older children, there are only changes in the kind of water contact activities, which may not necessarily translate to major change in rate of exposure to the disease. Surprisingly, subgroup analysis showed strong positive association between gender and reinfection with *S. japonicum*. Although this may be due to differences in gender role in various cultural settings, the observed association may not be very reliable since the analysis was based on only four studies on *S. japonicum*. Also on cultural differences, Fulford *et al.* (1996) and other workers identified that patterns of water contact vary dramatically between even culturally rather similar communities [57], [58]. Therefore, absence of strong association with gender even with apparent behavioral differences between genders remains inconclusive. This implies that gender difference in reinfection pattern varies in difference cultural settings [58].

Apart from the demographic factors, three major epidemiological factors were positively correlated with reinfection: high pre-treatment intensity, high rate of exposure and residence in high transmission area. High pretreatment intensity is related to possibility of failed or incomplete treatment [36], especially when studies did not include a follow up study dedicated to confirming cure in the treated population. As would be expected, there was also a positive correlation between reinfection and high exposure rate as inferred from the four studies assessing this factor, but the association was not statistically significant. Although some studies distinguished between high transmission areas and low transmission areas based on relative availability of potable water and sanitation [36], there was no association between residence in either of these areas, and rate of reinfection with schistosomes. This could be because water contact activities do not depend exclusively on lack of domestic water or sanitation, but is a function of several interrelated factors, including: perception, distinct cultural practices and the need for recreation. These render residence in either high or low transmission areas less important as a risk factor of reinfection with schistosomes.

Among the immunological factors identified, most studies reported association between different antibody isotype levels and probability of reinfection with schistosomes. Interestingly, a negative association was recorded from the association between IgE levels and reinfection with schistosomes. On the other hand, strong positive association was observed between IgG4 levels and reinfection with schistosomes. These observations are consistent with the consensus perspective that IgE/IgG4 balance plays central role in controlling protective immunity against infection or reinfection with schistosomes [2]–[5], [8], [31], [38], [39], [41], [44], [56], [59]–[63]. While increased IgE levels are protective against infection and reinfection, elevated IgG4 levels increase predisposition to infection and reinfection. Recent studies have found strong association between IgE levels and certain loci in the human genome, including: the cytokine gene cluster on chromosome 5q31-q33 (SM1), which also controls infection with schistosomiasis [7], [64]–[67]; *FCER1A* on chromosome 1q23, which is the gene encoding the alpha chain of the high affinity receptor for IgE [11], [14]; *STAT4* on chromosome 2q32 [12], [64] which controls Th1 development; *STAT6* on chromosome 12q13 [12], [13], [15], [64] and *GATA3* on chromosome 10p15 [12], [64] which control Th2 development; and the Th2 cytokine receptor cluster in 16p12 region of the human genome [7], [64]. We had expected to identify studies assessing association between reinfection and several immunogenetic factors including variations in these loci; however, there were few or no studies on the host immunogenetic factors of reinfection with schistosomes, an important theme for further research. We are presently proposing a study that will identify association between major single nucleotide polymorphisms (SNPs) in these loci, and reinfection with schistosomes and other helminthic infections.

Our analyses on cellular immune response factors showed negative effect estimates for the associations of reinfection with levels of IFN-γ, IL-5, IL-10, TNF-α and CD4^+^ T-helper cells; and positive effect estimates for the association of reinfection with the proportions of CD8^+^, CD19^+^ and CD16^+^ cells. However, these inferences are not very reliable since there was consistently limited number of studies reporting these host factors, and the associations were not statistically significant (Table 1 and Table S2). An interesting observation though is the negative effect estimate recorded from the association of reinfection with schistosomes with levels of IFN-γ. Studies in both human and animal models have shown that protective immunity against schistosomiasis is mainly Th1 dependent [2], [4], [6], [68]–[75]. The egg antigen drives a dominant Th2 response. Thus, induction and sustenance of a Th1 environment at the acute phase of infection thru onset of egg deposition is required for sterile and anti-pathology protection [68], [69], [75]–[78]. Negative effect estimates were also recorded from the association of reinfection with levels of IL-5 and IL-13. This is consistent with the notion that these cytokines control release and survival of eosinophil [31], which has been shown to induce antibody dependent protective immunity against schistosomiasis [79], [80]. Other factors including the HLA gene polymorphisms, levels of total protein, albumin, total cholesterol, low-density lipoproteins and very low-density lipoproteins were also identified. However, these factors were all reported by only one study (Table S2).

In conclusions, this study has identified the major host determinants of resistance or susceptibility to reinfection with schistosomes; although we had anticipated studies on immunogenetic factors in addition to the identified socio-demographic, epidemiological and immunological factors. Therefore, there is need to explore the association between reinfection with schistosomes and host immunogenetic factors, especially the variations in the genes controlling immune response against schistosomiasis. This will be an interesting subject for further studies. Strong association with age and water contact related factors has reaffirmed that these factors play dominant role in determining exposure to pathogen and disease transmission. We also reconfirmed the major role played by IgE/IgG4 balance in controlling protective immunity against infection and reinfection with schistosomes.

## Supporting Information

Figure S1 **The PRISMA 2009 checklist.** This study followed the guidelines of the PRISMA statement for conduct and reporting systematic reviews and meta-analyses.
(DOC)

Figure S2 **Association of IgE and IgG4 levels with reinfection with schistosomes (detailed).** This is the same data as shown in Figure 5 and Figure 6, except that the details of calculation of continuous variables are shown.
(PDF)

Figure S3 **Association of levels of anti-SWA antibody isotypes with reinfection with schistosomes.** Forest plots for the association of reinfection with IgG1 (**A**), IgG2 (**B**), IgG3 (**C**), IgA (**D**), and IgM (**E**).
(TIF)

Figure S4 **Association of levels of anti-SEA antibody isotypes with reinfection with schistosomes.** Forest plot for the association of reinfection with IgG1 (**A**), IgG2 (**B**), IgG3 (**C**), IgA (**D**), and IgM (**E**).
(TIF)

Table S1 **Characteristics of studies included in the meta-analysis of host factors of reinfection with schistosomes.**
(XLSX)

Table S2 **Summary of overall effect measures for host determinants of reinfection identified in this study.** This Table is similar to Table 1, but other host determinants reported by only one study are listed as well.
(DOC)

Table S3 **Sensitivity analysis by exclusion of individual studies or subgroups from the meta-analysis.**
(XLS)

Table S4 **Sensitivity analysis by sample size ordered cumulative meta-analysis for the association of age and gender with reinfection with schistosomes.**
(XLS)
