# Supplementary figures and images for: Host Determinants of Reinfection with Schistosomes in Humans: A Systematic Review and Meta-analysis
Source: PLoS Negl Trop Dis. 2014 Sep 11;8(9):e3164. doi: 10.1371/journal.pntd.0003164 (PMC4161334; doi:10.1371/journal.pntd.0003164)

## A. SWA IgE

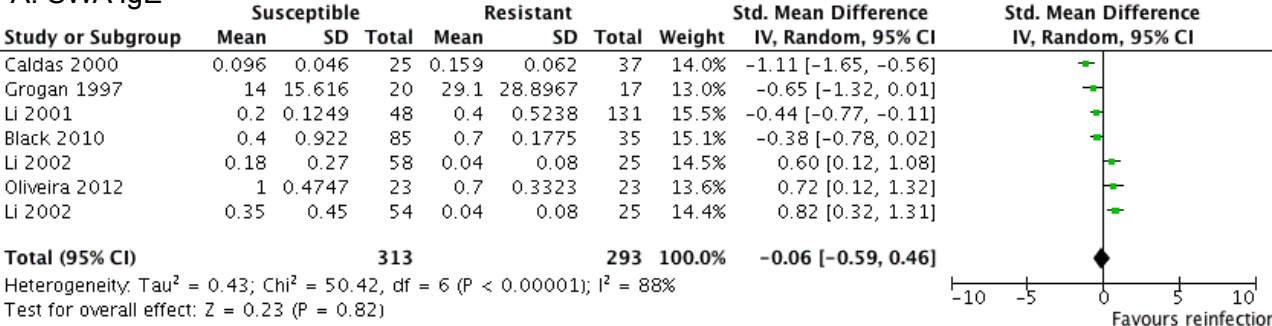

## B. SEA IgE

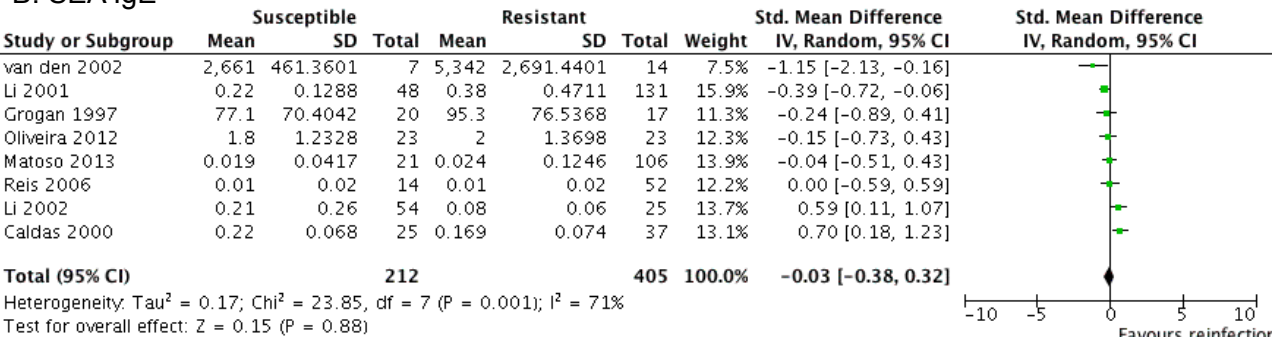

## C. SWA IgG4

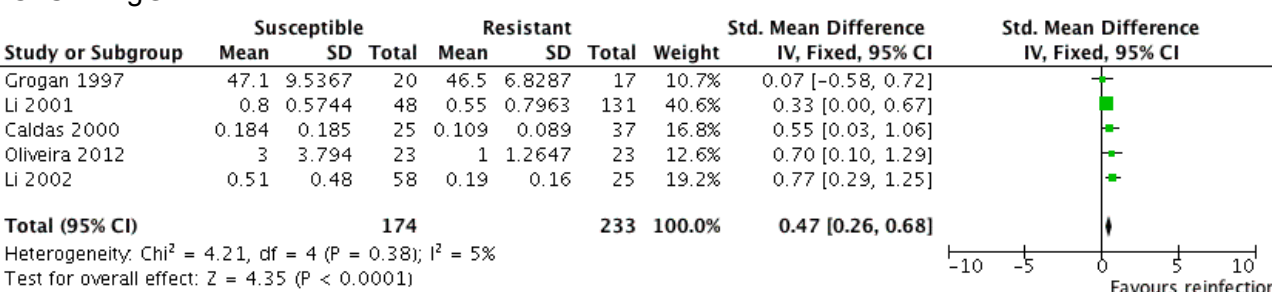

## D. SEA IgG4

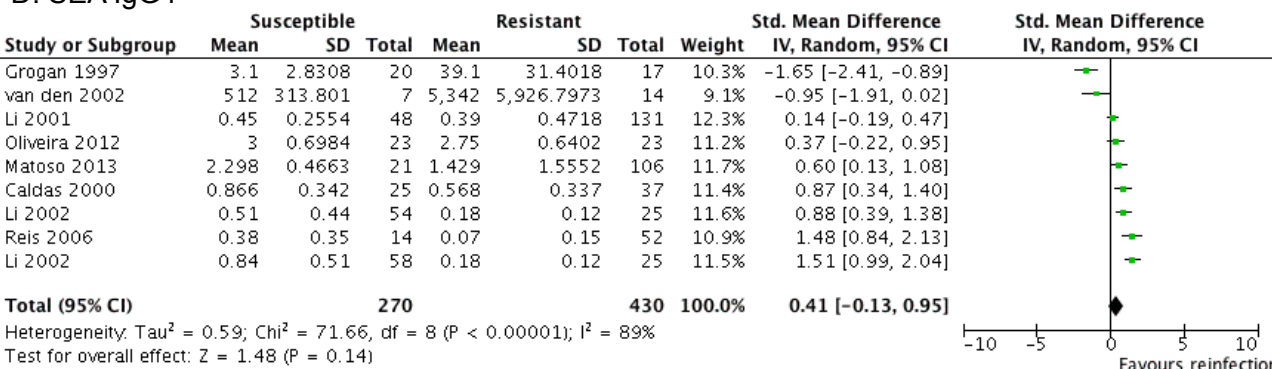

Supplement: Figure S2 — Association of IgE and IgG4 levels with reinfection with schistosomes (detailed). This is the same data as shown in Figure 5 and Figure 6, except that the details of calculation of continuous variables are shown. (PDF) [file pntd.0003164.s002.pdf]

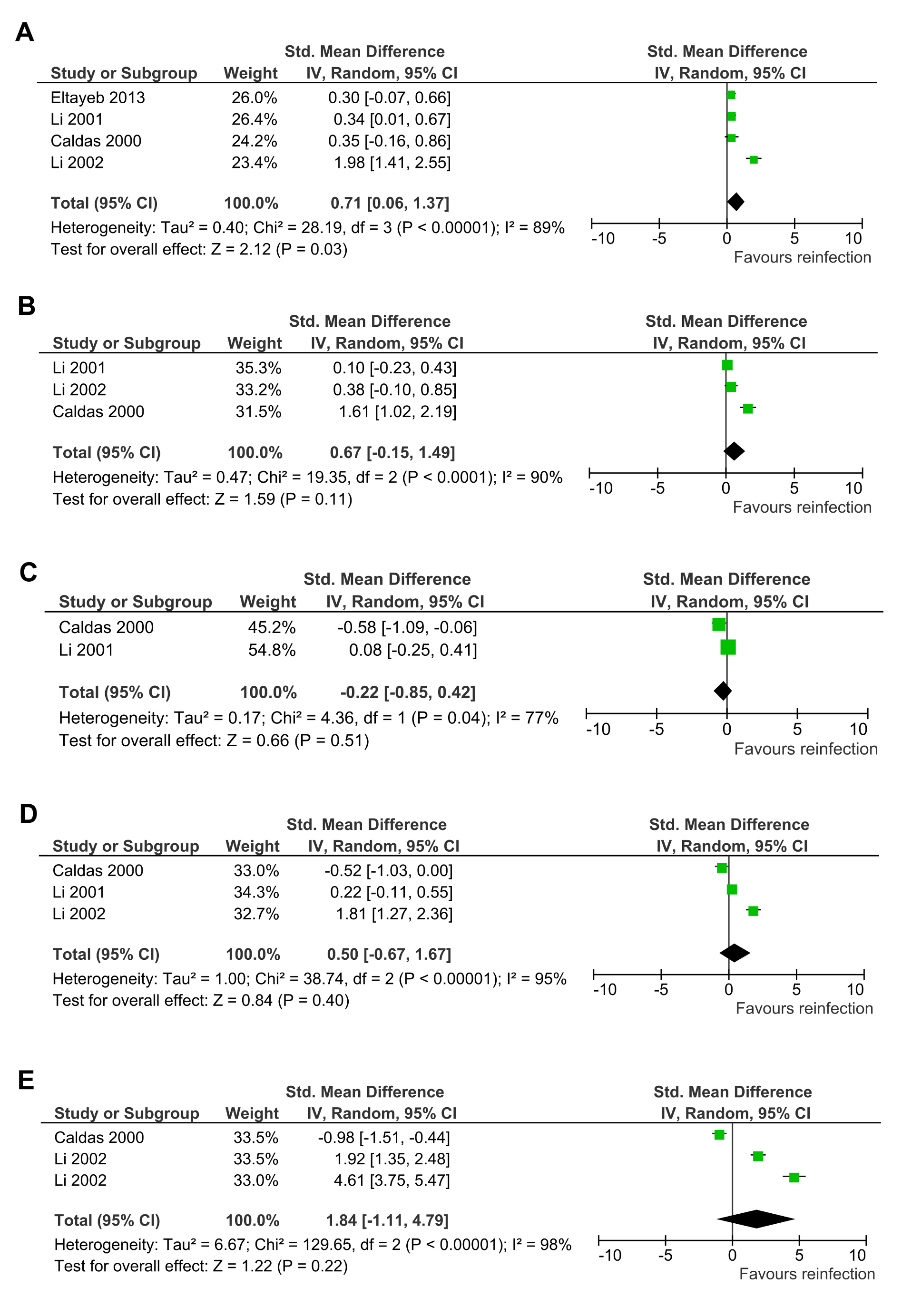

Supplement: Figure S3 — Association of levels of anti-SWA antibody isotypes with reinfection with schistosomes. Forest plots for the association of reinfection with IgG1 (A), IgG2 (B), IgG3 (C), IgA (D), and IgM (E). (TIF) [file pntd.0003164.s003.tif]

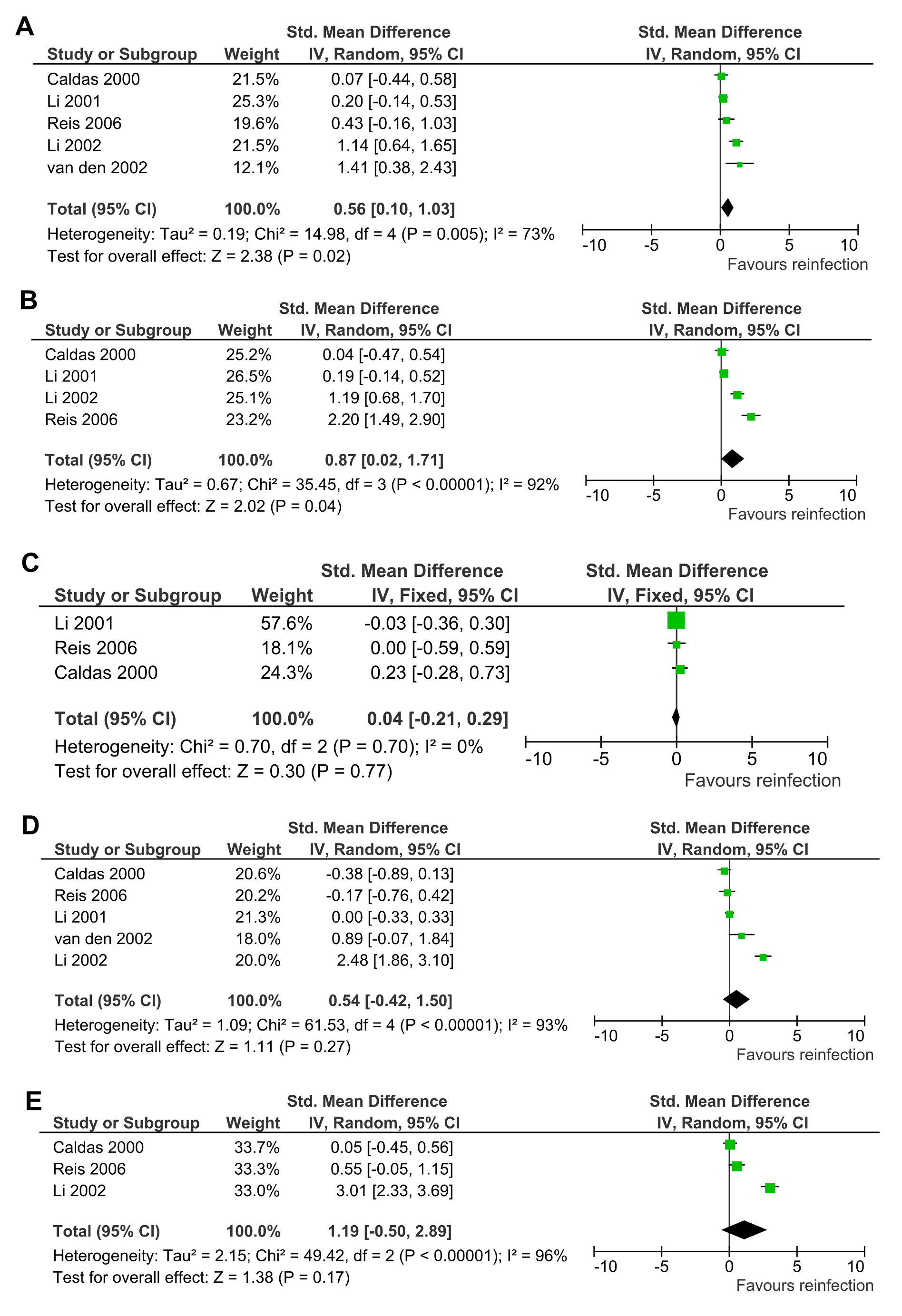

Supplement: Figure S4 — Association of levels of anti-SEA antibody isotypes with reinfection with schistosomes. Forest plot for the association of reinfection with IgG1 (A), IgG2 (B), IgG3 (C), IgA (D), and IgM (E). (TIF) [file pntd.0003164.s004.tif]
