# Supplementary material for: Host Determinants of Reinfection with Schistosomes in Humans: A Systematic Review and Meta-analysis
Source: PLoS Negl Trop Dis. 2014 Sep 11;8(9):e3164. doi: 10.1371/journal.pntd.0003164 (PMC4161334; doi:10.1371/journal.pntd.0003164)
Supplement: Table S2 — Summary of overall effect measures for host determinants of reinfection identified in this study. This Table is similar to Table 1, but other host determinants reported by only one study are listed as well. (DOC) [file pntd.0003164.s006.doc]

**Table S2. Host factors of reinfection with schistosomes identified in this study**

| **Host determinants** | **No of Studies** | **Model** | **Heterogeneity** | | **Association** | | **Study References** |  | | | | |
| --- | --- | --- | --- | --- | --- | --- | --- | --- | --- | --- | --- | --- |
|  |  |  | ***X^2^* (*p*-value)** | ***I^2^*** | **Pooled Risk Ratio (95% CI)** | ***p*-value** |  |  | | | | |
| 1. **Demographic factors** | | | | | | | |  | | | | |
| Age (< 10) | 19 | Random | *p* < 0.0001 | 74 | 1.91 [1.41, 2.60] | *p* < 0.0001 | [1-17] | |  | | | |
| Gender (Male) | 20 | Random | *p* < 0.0001 | 88 | 1.45 [1.02, 2.05] | *p* = 0.04 | [1-5,7,8,10-13,16-22] | |  | | | |
| 1. **Epidemiological factors** | | | | | | | |  | |  |  | 1. **Epidemiological factors** |
| Pre-treatment intensity | 7 | Random | *p* = 0.006 | 67 | 2.85 [1.97, 4.12] | *p*<0.0001 | [3,12,13,17,23] |  | | | | |
| High exposure | 4 | Random | *p* < 0.0001 | 91 | 2.34 [0.93, 5.85] | *p*=0.07 | [8,13,17,19] |  | | | | |
| High transmission area | 5 | Random | *p* < 0.0001 | 91 | 2.24 [0.63, 7.91] | *p*=0.21 | [8,14,19,24] |  | | | | |
| 1. **Antibodies** | | | | | | | |  | | | | |
| - **IgE** |  |  |  |  |  |  |  |  | | | | |
| SWA | 7 | Random | *p* < 0.0001 | 88 | -0.06 [-0.59, 0.46] | *p* = 0.82 | [4,16,19,25-27] |  | | | | |
| SEA | 8 | Random | *p* = 0.001 | 71 | -0.03 [-0.38, 0.32] | *p* = 0.88 | [4,16,19,25,27-30] |  | | | | |
| - **IgG4** |  |  |  |  |  |  |  |  | | | | |
| SWA | 5 | Fixed | *p* = 0.38 | 5 | 0.47 [0.26, 0.68] | *p* < 0.0001 | [4,16,19,25,27] |  | | | | |
| SEA | 9 | Random | *p* < 0.0001 | 89 | 0.41 [-0.13, 0.95] | *p* = 0.14 | [4,16,19,25,27-30] |  | | | | |
| - **IgG1** |  |  |  |  |  |  |  |  | | | | |
| SWA | 4 | Random | *p* < 0.0001 | 89 | 0.71 [0.06, 1.37] | *p* = 0.03 | [4,21,25,27] |  | | | | |
| SEA | 5 | Random | *p* = 0.005 | 73 | 0.56 [0.10, 1.03] | *p* = 0.02 | [4,25,27,28,30] |  | | | | |
| - **IgG2** |  |  |  |  |  |  |  |  | | | | |
| SWA | 3 | Random | *p* < 0.0001 | 90 | 0.67 [-0.15, 1.49] | *p* = 0.11 | [4,25,27] |  | | | | |
| SEA | 4 | Random | *p* < 0.0001 | 92 | 0.87 [0.02, 1.71] | *p* = 0.04 | [4,25,27,30] |  | | | | |
| - **IgG3** |  |  |  |  |  |  |  |  | | | | |
| SWA | 2 | Random | *p* = 0.04 | 77 | -0.22 [-0.85, 0.42] | *p* = 0.51 | [4,25] |  | | | | |
| SEA | 3 | Fixed | *p* = 0.70 | 0 | 0.04 [-0.21, 0.29] | *p* = 0.77 | [4,25,30] |  | | | | |
| - **IgA** |  |  |  |  |  |  |  |  | | | | |
| SWA | 3 | Random | *p* < 0.0001 | 95 | 0.50 [-0.67, 1.67] | *p* = 0.40 | [4,25,27] |  | | | | |
| SEA | 5 | Random | *p* < 0.0001 | 93 | 0.54 [-0.42, 1.50] | *p* = 0.27 | [4,25,27,28,30] |  | | | | |
| - **IgM** |  |  |  |  |  |  |  |  | | | | |
| SWA | 3 | Random | *p* < 0.0001 | 98 | 1.84 [-1.11, 4.79] | *p* = 0.22 | [4,25,27] |  | | | | |
| SEA | 3 | Random | *p* < 0.0001 | 96 | 1.19 [-0.50, 2.89] | *p* = 0.17 | [4,27,30] |  | | | | |
| 1. **Cytokines** | | | | | | | |  | | | | |
| IFN-g | 4 | Fixed | *p* = 0.36 | 1 | -0.22 [-0.52, 0.08] | *p* = 0.15 | [19,21,28,31] |  | | | | |
| IL-10 | 4 | Fixed | *p* = 0.08 | 56 | -0.15 [-0.44, 0.13] | *p* = 0.29 | [19,21,28,31] |  | | | | |
| TNF-a | 2 | Random | *p* = 0.03 | 79 | -0.27 [-0.77, 0.22] | *p* = 0.28 | [30,31] |  | | | | |
| IL-5 | 3 | Random | *p* = 0.004 | 82 | -0.17 [-1.38, 1.04] | *p* = 0.78 | [19,28,31] |  | | | | |
| IL-13 | 1 | N/A | N/A | N/A | -1.01 [-1.97, -0.04] | *p* = 0.04 | [28] |  | | | | |
| 1. **Immune cell surface marker** | | | | | | | |  | | | | |
| CD4 | 2 | Fixed | *p* = 0.25 | 23 | -0.62 [-1.05, -0.18] | *p* = 0.005 | [31,32] |  | | | | |
| CD8 | 2 | Random | *p* = 0.003 | 89 | 0.08 [-1.71, 1.86] | *p* = 0.93 | [31,32] |  | | | | |
| CD19 | 2 | Fixed | *p* = 0.40 | 0 | 0.38 [-0.04, 0.81] | *p* = 0.08 | [31,32] |  | | | | |
| CD16 | 1 | N/A | N/A | N/A | 0.66 [-0.39, 1.7] | *p* = 0.22 | [31] |  | | | | |
| 1. **Genetic Variants** | | | | | | | |  | | | | |
| HLA-DRB1 | 1 | N/A | N/A | N/A | N/A | N/A | [33] |  | | | | |
| HLA-DQA1 | 1 | N/A | N/A | N/A | N/A | N/A | [34] |  | | | | |
| HLA-DQB1 | 1 | N/A | N/A | N/A | N/A | N/A | [34] |  | | | | |
| HLA-DPA1 | 1 | N/A | N/A | N/A | N/A | N/A | [33] |  | | | | |
| HLA-DPB1 | 1 | N/A | N/A | N/A | N/A | N/A | [33] |  | | | | |
| MIC-A | 1 | N/A | N/A | N/A | N/A | N/A | [35] |  | | | | |
| 1. **Others** | | | | | | | |  | | | | |
| Total protein | 1 | N/A | N/A | N/A | N/A | N/A | [30] |  | | | | |
| Albumin | 1 | N/A | N/A | N/A | N/A | N/A | [30] |  | | | | |
| Total cholesterol | 1 | N/A | N/A | N/A | N/A | N/A | [30] |  | | | | |
| LDL | 1 | N/A | N/A | N/A | N/A | N/A | [30] |  | | | | |
| VLDL | 1 | N/A | N/A | N/A | N/A | N/A | [30] |  | | | | |

**References**

1. Pinot de Moira A, Fulford AJ, Kabatereine NB, Ouma JH, Booth M, et al. (2010) Analysis of complex patterns of human exposure and immunity to Schistosomiasis mansoni: the influence of age, sex, ethnicity and IgE. PLoS Negl Trop Dis 4.

2. Pinot de Moira A, Jones FM, Wilson S, Tukahebwa E, Fitzsimmons CM, et al. (2013) Effects of treatment on IgE responses against parasite allergen-like proteins and immunity to reinfection in childhood schistosome and hookworm coinfections. Infect Immun 81: 23-32.

3. Tukahebwa EM, Vennervald BJ, Nuwaha F, Kabatereine NB, Magnussen P (2013) Comparative efficacy of one versus two doses of praziquantel on cure rate of Schistosoma mansoni infection and re-infection in Mayuge District, Uganda. Trans R Soc Trop Med Hyg 107: 397-404.

4. Caldas IR, Correa-Oliveira R, Colosimo E, Carvalho OS, Massara CL, et al. (2000) Susceptibility and resistance to Schistosoma mansoni reinfection: parallel cellular and isotypic immunologic assessment. Am J Trop Med Hyg 62: 57-64.

5. Roberts M, Butterworth AE, Kimani G, Kamau T, Fulford AJ, et al. (1993) Immunity after treatment of human schistosomiasis: association between cellular responses and resistance to reinfection. Infect Immun 61: 4984-4993.

6. Zhang Z, Wu H, Chen S, Hu L, Xie Z, et al. (1997) Association between IgE antibody against soluble egg antigen and resistance to reinfection with Schistosoma japonicum. Trans R Soc Trop Med Hyg 91: 606-608.

7. Acosta LP, Aligui GD, Tiu WU, McManus DP, Olveda RM (2002) Immune correlate study on human Schistosoma japonicum in a well-defined population in Leyte, Philippines: I. Assessment of 'resistance' versus 'susceptibility' to S. japonicum infection. Acta Trop 84: 127-136.

8. Wu Z, Bu K, Yuan L, Yang G, Zhu J, et al. (1993) Factors contributing to reinfection with schistosomiasis japonica after treatment in the lake region of China. Acta Trop 54: 83-88.

9. Medhat A, Shehata M, Bucci K, Mohamed S, Dief AD, et al. (1998) Increased interleukin-4 and interleukin-5 production in response to Schistosoma haematobium adult worm antigens correlates with lack of reinfection after treatment. J Infect Dis 178: 512-519.

10. Saathoff E, Olsen A, Magnussen P, Kvalsvig JD, Becker W, et al. (2004) Patterns of Schistosoma haematobium infection, impact of praziquantel treatment and re-infection after treatment in a cohort of schoolchildren from rural KwaZulu-Natal/South Africa. BMC Infect Dis 4: 40.

11. Ofoezie IE (2000) Patterns of reinfection following praziquantel treatment of urinary schistosomiasis at a period of low transmission. Acta Trop 75: 123-126.

12. King CH, Lombardi G, Lombardi C, Greenblatt R, Hodder S, et al. (1988) Chemotherapy-based control of schistosomiasis haematobia. I. Metrifonate versus praziquantel in control of intensity and prevalence of infection. Am J Trop Med Hyg 39: 295-305.

13. Satayathum SA, Muchiri EM, Ouma JH, Whalen CC, King CH (2006) Factors affecting infection or reinfection with Schistosoma haematobium in coastal Kenya: survival analysis during a nine-year, school-based treatment program. Am J Trop Med Hyg 75: 83-92.

14. Mutapi F, Ndhlovu PD, Hagan P, Woolhouse ME (1999) A comparison of re-infection rates with Schistosoma haematobium following chemotherapy in areas with high and low levels of infection. Parasite Immunol 21: 253-259.

15. Hagan P, Blumenthal UJ, Dunn D, Simpson AJ, Wilkins HA (1991) Human IgE, IgG4 and resistance to reinfection with Schistosoma haematobium. Nature 349: 243-245.

16. Grogan JL, Kremsner PG, van Dam GJ, Deelder AM, Yazdanbakhsh M (1997) Anti-schistosome IgG4 and IgE at 2 years after chemotherapy: infected versus uninfected individuals. J Infect Dis 176: 1344-1350.

17. Etard JF, Audibert M, Dabo A (1995) Age-acquired resistance and predisposition to reinfection with Schistosoma haematobium after treatment with praziquantel in Mali. Am J Trop Med Hyg 52: 549-558.

18. Fonseca CT, Cunha-Neto E, Goldberg AC, Kalil J, de Jesus AR, et al. (2005) Identification of paramyosin T cell epitopes associated with human resistance to Schistosoma mansoni reinfection. Clin Exp Immunol 142: 539-547.

19. Oliveira RR, Figueiredo JP, Cardoso LS, Jabar RL, Souza RP, et al. (2012) Factors associated with resistance to Schistosoma mansoni infection in an endemic area of Bahia, Brazil. Am J Trop Med Hyg 86: 296-305.

20. Gundersen SG, Birrie H, Torvik HP, Medhin G, Mengesha H (1998) Delayed reinfection of Schistosoma mansoni in the Blue Nile Valley of western Ethiopia 10 years after mass chemotherapy. Acta Trop 70: 35-42.

21. Eltayeb NM, Mukhtar MM, Mohamed AB (2013) Epidemiology of schistosomiasis in Gezira area Central Sudan and analysis of cytokine profiles. Asian Pac J Trop Med 6: 119-125.

22. Li YS, Sleigh AC, Ross AG, Li Y, Williams GM, et al. (1999) A 2-year prospective study in China provides epidemiological evidence for resistance in humans to re-infection with Schistosoma japonicum. Ann Trop Med Parasitol 93: 629-642.

23. Etard JF, Borel E, Segala C (1990) Schistosoma haematobium infection in Mauritania: two years of follow-up after a targeted chemotherapy--a life-table approach of the risk of reinfection. Parasitology 100 Pt 3: 399-406.

24. Garba A, Lamine MS, Barkire N, Djibo A, Sofo B, et al. (2013) Efficacy and safety of two closely spaced doses of praziquantel against Schistosoma haematobium and S. mansoni and re-infection patterns in school-aged children in Niger. Acta Trop 128: 334-344.

25. Li Y, Sleigh AC, Ross AG, Zhang X, Williams GM, et al. (2001) Human susceptibility to Schistosoma japonicum in China correlates with antibody isotypes to native antigens. Trans R Soc Trop Med Hyg 95: 441-448.

26. Black CL, Mwinzi PN, Muok EM, Abudho B, Fitzsimmons CM, et al. (2010) Influence of exposure history on the immunology and development of resistance to human Schistosomiasis mansoni. PLoS Negl Trop Dis 4: e637.

27. Li Y, Yu DB, Li YS, Luo XS, Liang YS, et al. (2002) Antibody isotype responses to Schistosoma japonicum antigens in subjects from a schistosomiasis area with repeated praziquantel chemotherapy compared with a new endemic zone in Hunan Province, P.R. China. Trans R Soc Trop Med Hyg 96: 210-215.

28. van den Biggelaar AH, Borrmann S, Kremsner P, Yazdanbakhsh M (2002) Immune responses induced by repeated treatment do not result in protective immunity to Schistosoma haematobium: interleukin (IL)-5 and IL-10 responses. J Infect Dis 186: 1474-1482.

29. Matoso LF, Oliveira-Prado R, Abreu MN, Fujiwara RT, Loverde PT, et al. (2013) Longitudinal analysis of antigen specific response in individuals with Schistosoma mansoni infection in an endemic area of Minas Gerais, Brazil. Trans R Soc Trop Med Hyg.

30. Reis EA, Reis MG, Silva Rde C, Carmo TM, Assis AM, et al. (2006) Biochemical and immunologic predictors of efficacy of treatment or reinfection risk for Schistosoma mansoni. Am J Trop Med Hyg 75: 904-909.

31. Brito CF, Caldas IR, Coura Filho P, Correa-Oliveira R, Oliveira SC (2000) CD4+ T cells of schistosomiasis naturally resistant individuals living in an endemic area produce interferon-gamma and tumour necrosis factor-alpha in response to the recombinant 14KDA Schistosoma mansoni fatty acid-binding protein. Scand J Immunol 51: 595-601.

32. Martins-Filho OA, Cunha-Melo JR, Lambertucci JR, Silveira AM, Colley DG, et al. (1999) Clinical forms of human Schistosoma mansoni infection are associated with differential activation of T-cell subsets and costimulatory molecules. Dig Dis Sci 44: 570-577.

33. Booth M, Shaw MA, Carpenter D, Joseph S, Kabatereine NB, et al. (2006) Carriage of DRB1*13 is associated with increased posttreatment IgE levels against Schistosoma mansoni antigens and lower long-term reinfection levels. J Immunol 176: 7112-7118.

34. May J, Kremsner PG, Milovanovic D, Schnittger L, Loliger CC, et al. (1998) HLA-DP control of human Schistosoma haematobium infection. Am J Trop Med Hyg 59: 302-306.

35. Gong Z, Luo QZ, Lin L, Su YP, Peng HB, et al. (2012) Association of MICA gene polymorphisms with liver fibrosis in schistosomiasis patients in the Dongting Lake region. Braz J Med Biol Res 45: 222-229.
